# Radicular Cyst associated with Deciduous Incisor: A Rare Case Report

**DOI:** 10.5005/jp-journals-10005-1170

**Published:** 2012-12-05

**Authors:** P Latha Subramanya

**Affiliations:** Reader, Department of Pedodontics, TODC, Karnataka, India e-mail: drlatas@yahoo.co.in

**Keywords:** Radicular cyst, Primary tooth, Deciduous

## Abstract

Radicular cysts are considered rare in the primary dentition, comprising only 0.5 to 3.3% of the total number of radicular cysts in both primary and permanent dentitions. The aim of this case report is to present the clinical, radiographic and histological characteristics of radicular cyst associated with primary central incisor. Extraction and enucleation of the cyst was carried out under local anesthesia after elevation of the mucoperiosteal flap, which led to uneventful healing.

**How to cite this article:** Subramanya PL. Radicular Cyst associated with Deciduous Incisor: A Rare Case Report. Int J Clin Pediatr Dent 2012;5(3):217-219.

## INTRODUCTION

Children exhibit many pathological lesions involving the jaw bones. Most common among these lesions of inflammatory origin is radicular cyst, which are common sequelae of dental caries. Radicular cysts are odontogenic cysts which are derived from the inflammatory activation of epithelial root sheath residues (cell rests of Malassez). Radicular cysts are considered to be rare in the primary dentition^1^ comprising only 0.5 to 3.3% of the total number of radicular cysts in both the primary and permanent dentition.^1,2^ Radicular cysts are usually asymptomatic and are left unnoticed, until detected by routine radiography.^3^

## CASE REPORT

**Fig. 1:**
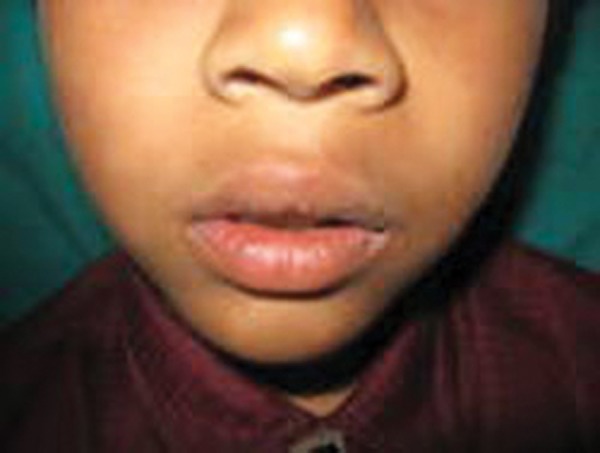
Clinical photograph showing raised ala of the nose on the left side

**Fig. 2:**
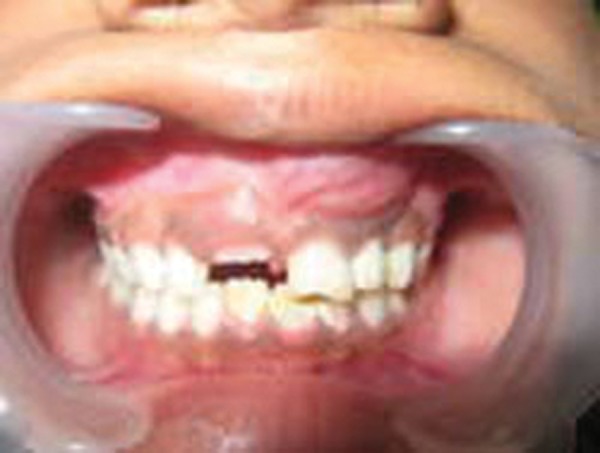
Clinical photograph depicting swelling in relation to left deciduous incisors

**Fig. 3:**
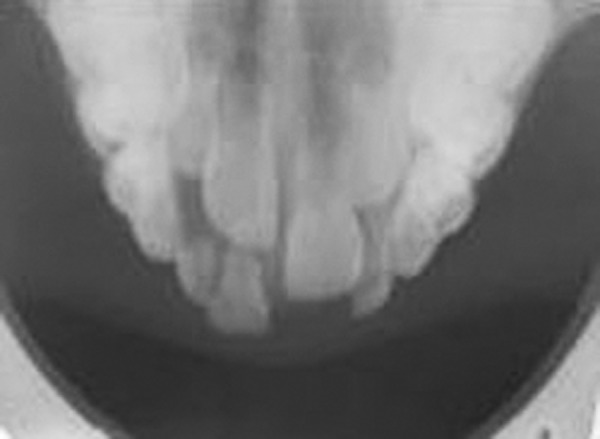
Intraoral occlusal radiograph showing radiolucency in relation to the apices of left deciduous incisors

**Fig. 4:**
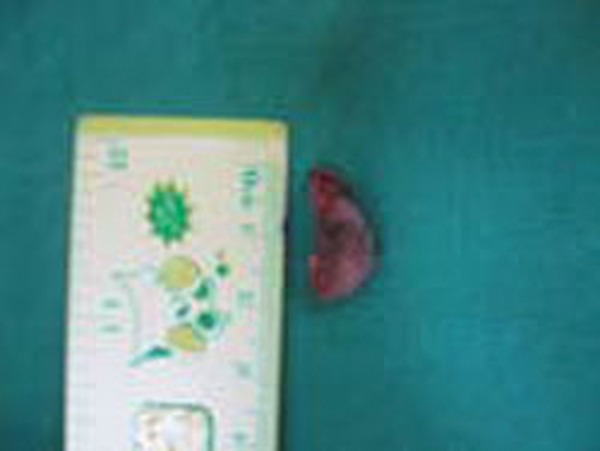
Enucleated cyst

**Figs 5A and B:**
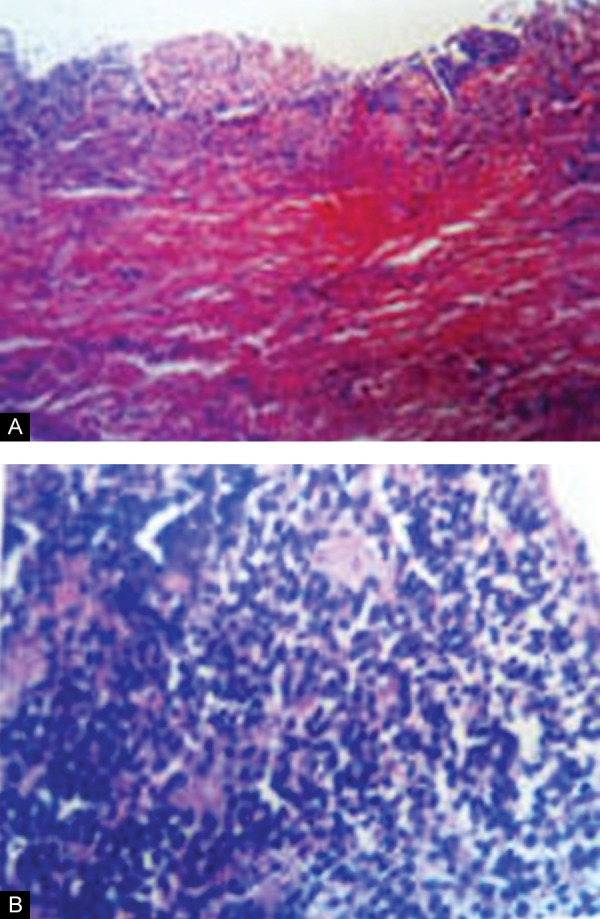
Histopathological slide of the cystic lesion

**Figs 6A and B:**
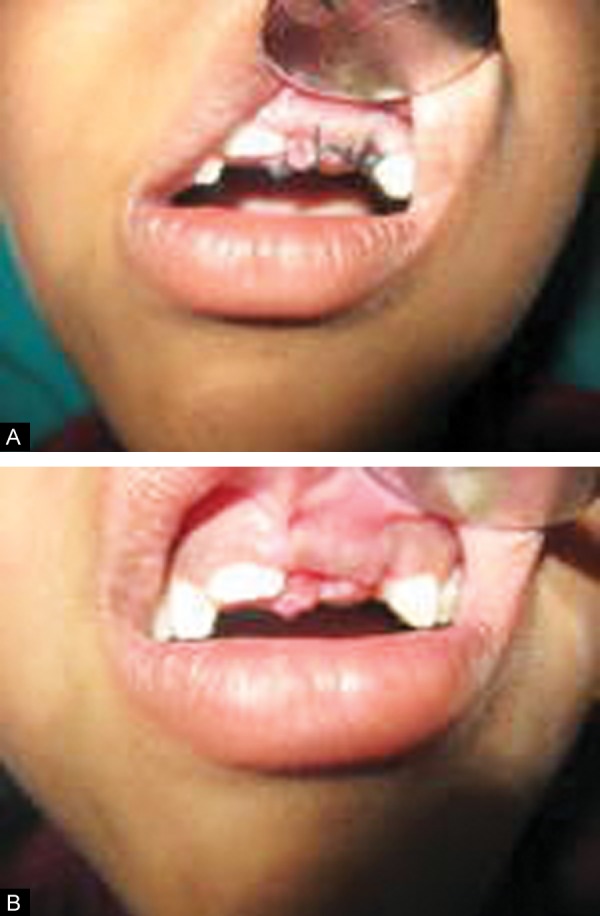
Postoperative clinical photographs

An 8-year-old boy presented with a 15 days history of an asymptomatic swelling below the alla of the nose on the left side (Fig. 1). Patients past dental history indicated trauma with respect to 61 about 2 years ago. Extraorally, the swelling was diffuse, nontender and bony hard. Intraorally, an irregular bony hard swelling extending from the left maxillary primary incisor to canine area was seen. The left maxillary primary incisor, i.e. 61 exhibited fracture of the incisal edge involving dentine (Fig. 2). The differential diagnosis being radicular cyst periapical cyst, traumatic bone cyst, globulomaxillary cyst and aneurysmal bone cyst. Radiographs revealed well-defined large periapical radiolucency with a thin sclerotic border in relation to apex of 61 (Fig. 3). Root of deciduous left central incisor was resorbed. From history and clinical presentation, a provisional diagnosis of radicular cyst was made. The cystic site was exposed under local anesthesia after elevation of the mucoperiosteal flap, which exhibited expansion and thinning of buccal cortical plate. The cyst was enucleated along with the extraction of 61 (Fig. 4). The specimen was sent for histopathologic examination. Surgical exploration confirmed the nonassociation of the cyst to the successive permanent teeth. Primary closure was done following debridement and hemostasis. Postsurgical healing was uneventful (Figs 6A and B). Microscopically, a densely inflamed cyst wall covered by a varying thickness of nonkeratinized epithelial lining suggested radicular cyst (Figs 5A and B).

## DISCUSSION

A radicular cyst is one which arises from the epithelial residues in the periodontal ligament as a result of inflammation. The inflammation usually follows the death of the dental pulp and cysts arising in this way are found most commonly at the apices of the involved teeth. Most of the radicular cysts are symptomless and are discovered during routine radiographic investigation.^1^ They are usually encountered in association with permanent teeth; however, occurrence in relation to deciduous teeth seems to be very rare.^4^ Radicular cysts arising from deciduous teeth are reported to occur in age range of 3 to 19 years with a male preponderance. The most commonly involved deciduous teeth are mandibular molars (67%), maxillary molars (17%) followed by anterior teeth.^5^ Very few cases of radicular cysts are seen in the first decade after which there is fairly a steep rise with a peak frequency in third decade. Radicular cysts arising from deciduous teeth are very less. Over 25 years period, of 1, 3000 radicular cysts only seven (0.5%) were associated with deciduous teeth.^1^ While Lustmann et al^6^ in an extensive review from 1898 to 1985, found only 28 cases to which they added 23 cases. Nagata et al^7^ in their review, report that there were 112 cases reported through 2004. Various reasons cited for this relative rarity include presence of deciduous teeth for a short time, easy drainage in deciduous teeth due to the presence of numerous accessory canals and a radicular radiolucency in relation to deciduous teeth are usually neglected. Additionally, the lesions tend to resolve on their own following the extraction/ exfoliation of the associated tooth and are generally not submitted for histopathological examination.^1,4^ Histologically, there is no difference between the cysts of primary teeth and those of permanent teeth except for rarity of cholesterol crystal slits in primary teeth cysts. This is due to the fact that the lesion associated with the primary teeth exists for shorter duration before removal in comparison to permanent teeth.^5^ Pulpal and periapical infections in deciduous teeth tend to drain more readily than those of permanent teeth and the antigenic stimuli, which evoke the changes leading to formation of radicular cysts, may be different.^8^

In the present case the treatment plan included extraction of the affected tooth followed by enucleation of the cyst. The other conservative and effective alternative mentioned being marsupialization of lesion with a fixed resin tube placed in alveolar hole after the extraction of the affected deciduous tooth.^9^ In a similar case the treatment comprised extraction of the primary teeth involved followed by marsupialization. A removable appliance with a resin extension into the cystic cavity was used to decompress the lesion. This treatment allowed rapid healing of the lesion and eruption of the permanent incisors without the need.^10^

The sequelae of an untreated or undiagnosed radicular cyst could be harmful to the patient’s future dental development. A patient with an untreated radicular cyst may present with swelling, tenderness, tooth mobility and a bluish tinge caused by buccal expansion of the cortical plates. Furthermore, displacement of the successor tooth or even more unforgiving, the loss of its vitality may result. However, if early diagnosis via regular radiographic monitoring of these teeth can be conducted, then it may prove productive in preventing the above problems, as well as the need for invasive surgical treatment.^11^ In children, healing of the postsurgical osseous defects is always good as they have high propensity for bone regeneration thus making post surgical healing uneventful.^1^

## CONCLUSION

This article presents a rare case of radicular cyst associated with deciduous incisor. Recognition of the sequelae of an untreated or undiagnosed radicular cyst is important for prevention of adverse effects to the underlying permanent successor as well as the need for invasive surgical treatment.
